# Addition of Orange Peel in Orange Jam: Evaluation of Sensory, Physicochemical, and Nutritional Characteristics

**DOI:** 10.3390/molecules25071670

**Published:** 2020-04-04

**Authors:** Flavia Teixeira, Bruna Aparecida dos Santos, Graziela Nunes, Jaqueline Machado Soares, Luane Aparecida do Amaral, Gabriel Henrique Oliveira de Souza, Juliano Tadeu Vilela de Resende, Bruna Menegassi, Bruna Paola Murino Rafacho, Kélin Schwarz, Elisvânia Freitas dos Santos, Daiana Novello

**Affiliations:** 1 Postgraduate Program Interdisciplinary in Community Development, State University of Midwest, 85040-167 Guarapuava, Brazil; teixeiraflavia19@gmail.com (F.T.); grazielaznunes@hotmail.com (G.N.); jaquue.s@gmail.com (J.M.S.); 2 Department of Nutrition, State University of Midwest, 85040-167 Guarapuava, Brazil; bruna.apsantos97@gmail.com; 3 Postgraduate Program in Health and Development in the Midwest Region, Medical School, Federal University of Mato Grosso do Sul, 79070-900 Campo Grande, Brazil; luapamaral@hotmail.com (L.A.d.A.); elisvania@gmail.com (E.F.d.S.); 4 Faculty of Pharmaceutical Sciences, Food and Nutrition, Federal University of Mato Grosso do Sul, 79070-900 Campo Grande, Brazil; g.henriqueoliveirasouza@gmail.com (G.H.O.d.S.); brunapaola@gmail.com (B.P.M.R.); 5 Department of Agronomy, State University of Londrina, 86057-970 Londrina, Brazil; jvresende@uel.br; 6 Faculty of Health Sciences, Federal University of Grande Dourados, 79825-070 Dourados, Brazil; brunamenegassi.nut@gmail.com; 7 Department of Nutrition, Federal University of Triângulo Mineiro, 38025-350 Uberaba, Brazil; kelinschwarz@hotmail.com

**Keywords:** by-products, *Citrus*, jam, orange, sensory attributes, bioactive compounds, food reuse, sweets

## Abstract

Orange is highly nutritious and a source of phytochemical compounds. However, its by-products are usually discarded. In this study, we evaluated the effect of orange peel (OP) addition in orange jam on sensory, physicochemical, and nutritional characteristics. Four jam formulations were elaborated with different OP levels: OP0 (standard), OP4, OP8, and OP12 (Orange Peel 0, 4, 8 and 12%, respectively). All samples were evaluated for sensory acceptability, and physicochemical and nutritional composition. The addition of 12% orange peel in jam reduced (*p* < 0.05) the acceptability for all evaluated attributes, as well as overall acceptance and purchase intention. However, OP utilization increased (*p* < 0.05) the levels of water activity, soluble solids, titratable acidity, and sugars. Soluble solids/titratable acidity ratio, luminosity (L*), and yellow content (b*) decreased in all added OP jams, while red content (a*) increased. No change in the pH and moisture values of the product were observed after OP addition. Ash, protein, lipid, dietary fiber, ascorbic acid, carotenoids, phenolic compounds, and antioxidant capacity values increased after OP addition, while carbohydrate and energy content decreased. A texture test showed that adhesiveness decreased, while gumminess, chewiness, and elasticity increased after OP addition. We concluded that the addition of up to 8% orange peel in jam maintains sensory acceptability similar to that of the standard product. OP addition is a viable alternative to improve some of the product’s physicochemical and nutritional characteristics.

## 1. Introduction

The food industry, consumers and researchers are showing increased interest in the use of unconventional ingredients in food products. This is attributed to the favorable nutritional profile of fruit and vegetable by-products, which may reduce the risk of developing non-communicable chronic diseases [1]. Food residues such as peel, seeds, membranes, stems, and leaves have a high rate of waste worldwide. In high-income countries, the disposal of by-products is around 30%, while in low-income countries, it surpasses 60% [2]. Brazil, for example, discards around 40–50% of by-products per year [3]. Food losses occur during production, processing, retailing, or due to incorrect disposal of restaurants and final consumers [4]. Nevertheless, peels, membranes, and seeds contain high nutritional content, suggesting a potential for addition as an ingredient in food products.

Fruits of the genus *Citrus* have seen a constant increase in commercial demand worldwide. In 2016, *Citrus* production reached 124.246 thousand tons [5]. Among them, orange stands out, with global production of 66.974 thousand tons/year. Brazil produces the most oranges, with cultivation of 14.350 thousand tons/year, followed by China with 7.000 thousand tons/year, India with 6.850 thousand tons/year, and the United States with 5.371 thousand tons/year [5]. The countries with the highest orange consumption are Luxembourg (104.76 kg/capita/year), Ireland (104.76 kg/capita/year), Belize (88.84 kg/capita/year), Netherlands (87.35 kg/capita/year), and Dominica (83.14 kg/capita/year). In Brazil, the annual consumption is 16.59 kg/capita [6].

Orange is a highly nutritious food, a source of phytochemical compounds like vitamin C, flavonoids, and carotenoids, which also give it its antioxidant property. It is commonly consumed fresh and in juices [7], jams [8], extracts for herbal medicines [9], and dietary supplements [10]. Its by-products (peel, membranes, and seeds) are generally disposed of in the environment, increasing the amount of organic waste in nature. The industry uses very little of the waste for the production of pectin, molasses, fibers, oils [11], and animal food [12]. Nevertheless, these by-products contain high levels of vitamin C [13], thiamine, niacin, pyridoxine, phosphorus, calcium, iron, magnesium, and potassium [14], as well as soluble and insoluble dietary fibers [15]. Fruit residues are also sources of bioactive compounds, especially flavonoids and phenolic acids [16]. Previous research has shown the positive effects of adding *Citrus* fruit peel to several products such as crackers [17], meatballs [18], marmalade [19], jam [20,21], and yogurt [22]. In a study by Younis et al. [20], the addition of sweet lemon peel in jam increased firmness and chewability, thus improving quality. However, stickiness, cohesiveness, and scores for texture, color, appearance, taste, and overall acceptance were reduced. In marmalade, the use of sweet orange peel powder increased phenolic compound content and antioxidant capacity, although there was a reduction in pH, titratable acidity, and anthocyanin content [19]. Thus, using *Citrus* fruit residues in food products might be viable for sensory, physicochemical, and nutritional profile improvements of foods, contributing to preservation of the environment.

Jams are a product with high acceptance. France has the largest jam production and intake in the world. In 2016, about 4 thousand tons were produced and 3.36 billion were consumed [23]. Other countries stand out in the production of jam; they include Turkey, Spain, Chile, India, China, the United States, and Brazil. In Brazil, the jam production reached 15.5 million tons in 2017 [24]. In general, it is a sweet made from fruit pulp, sugar, pectin, and citric acid [25]. The most produced and consumed jams are those made with grapes, apricots, blueberries [26], mangoes, pineapples [27], strawberries, oranges [25], and pomegranates [28]. Jams have low cost of production, are easy to process, transport, and store. Furthermore, they present an extended shelf life, as well as increase fruit utilization in the off-season. However, jams are produced only with pulp, leading to disposal of by-products. Thus, the aim of this research was to evaluate the effect of the addition of orange peel (OP) in orange jam on sensory, physicochemical, and nutritional characteristics.

## 2. Results and Discussion

### 2.1. Sensory Evaluation

Table 1 describes the sensory scores of orange jam added with different OP contents. The addition of 12% orange peel reduced (*p* < 0.05) the acceptability for appearance, aroma, taste, color, and overall acceptance attributes. Higher scores for texture and purchase intent were verified for OP0 and OP4 jam compared to OP12. The samples OP8 and OP12 did not present significant difference between them (*p* > 0.05). Similar results were found in papaya jam added with lemon peel (2.5 to 12.5%) [20]. Compounds present in OP such as poncirin, hesperidin, and polymethoxylated flavones are mainly responsible for the change in flavor of the jam [29,30].

In addition, soluble and volatile substances such as monoterpenes (limonene, mircene), sesquiterpenoids (α and β-sinensal), and sesquiterpene (valencene) [31] may alter product aroma. Attributes such as appearance and color can be altered by carotenoids present in OP, sine their pigmentation ranges from yellow to red. It has been reported that color changes might reduce consumer acceptance [32]. The OP fiber content was mainly responsible for the change in texture of the jam. Fibers increase the liquid retention capacity, promoting gelation networks and increasing jam consistency [33]. Despite lower jam grades with the addition of 12% orange peel, all formulations presented Acceptability Index (AI) > 70%, meaning good sensory acceptability [34].

### 2.2. Physicochemical and Nutritional Composition

The mean physicochemical composition of orange pulp and peel, and four jams is shown in Table 2. Higher pH (*p* < 0.05) was observed in the peel compared to the pulp, since the degradation of acids, especially citric acid, occurs after harvest. Acids are used by the cell structure as a respiratory substrate and/or for amino acid synthesis [35]. Higher Water activity (Aw), Soluble Solid (SS), Titratable Acidity (TA), SS/TA ratio, Non-Reducing Sugar (NRS), and Total Sugar (TS) contents were verified for the pulp compared to the peel. There was no statistical difference (*p* > 0.05) between the Reducing Sugar (RS) contents of the pulp and peel. The pulp contains higher concentrations of SS, NRS, and TS as it is a precursor of organic acid metabolism, photosynthesis, energy metabolism, and fruit cell structure [36]. 

The peel has lower amounts of TS as it protects the fruit from physical, chemical, and biological damage. Thus, it contains higher concentrations of essential oils [37]. TS include RS (glucose and fructose), NRS (sucrose), starch, and cellulose. In addition to having an important functional role in the structure and cellular function of orange pulp and peel, they are also responsible for the taste and aroma of the fruit [38].

Jams pH did not differ from each other (*p* > 0.05), which guarantees the stability of the product [25]. The addition of OP increased Aw, SS, TA, RS, NRS, and TS (*p* < 0.05). This effect can be attributed to the higher pectin content of OP (86.4 g 100 g^−1^) [39] when compared to pulp (42.25 g 100 g^−1^) [40]. During cooking, pectin carboxyl groups, which are in the form of free acids, bind to sugars and citric acid forming hydrogen bridges. This process origins a three-dimensional network that holds water, which increases SS concentration [41,42]. In addition, heat acts on sucrose, causing the loss of a water molecule, forming glucose and fructose [43]. TA determines the concentration of organic acids, which may influence the taste and aroma of foods. In general, products with lower acidity may have higher acceptability [44], an effect verified in the present research (Table 1). Higher SS/TA ratio was observed in standard jam, since the addition of OP reduced this parameter (*p* < 0.05). According to Zeliou et al. [45], higher SS/TA ratio indicates a balance between sugars and acids present in the food, which improves taste.

Color parameters L*, a* and b* of jam with different OP concentrations are shown in Table 3. Higher OP levels reduced lightness (L*) and yellow (b*), and increased red content (a*). In general, OP-added jam may be considered dark in color, as all L* values were less than 50%, with a yellow tone (b*) and a red tone (a*) [46]. These results are explained by the presence of chlorophyll and carotenoids in orange peel. In the presence of acids, chlorophyll is degraded to brown green pigments. In addition, carotenoids undergo auto-oxidation, especially at high temperatures, resulting in compounds with reddish tones [43]. In the sensory analysis, consumers noticed a difference in the color of the highest OP jam (12%), indicating a preference for jam with brighter and yellow tones.

The texture parameters of the jams are shown in Table 4. The addition of 12% orange peel reduced adhesiveness of the orange jam. Similar results were found for papaya jam added with lemon peel (2.5 to 12.5%) [20]. The viscosity and cohesiveness parameters did not change with OP addition (*p* > 0.05). However, there was a proportional increase (*p* < 0.05) in jam chewiness and gumminess, as higher OP contents were added. The OP8 and OP12 jam presented higher elasticity (*p* < 0.05), when compared to the OP0 and OP4 jam. The addition of OP increased the amount of pectin, forming a more rigid, elastic, and less-adhesive gel network [47]. In addition, orange peel presents higher fiber content, which increases water retention capacity by increasing chew ability [33]. Elasticity is also affected by OP acidity, leading to stiffness of pectin fibers, which in turn hardens the jam due to excessive pectin hydrolysis [47]. These changes in the instrumental texture profile confirm the lower acceptability of jam containing 12% orange peel, as it presented higher chewiness and lower adhesiveness. 

Table 5 presents the nutritional composition of orange pulp and peel, and four jam formulations. The pulp presented higher moisture content (*p* < 0.05) compared to the peel, corroborating with the results of Barros et al. [48], who evaluated five orange varieties grown in Goiás State, Brazil. Generally, fruits have high water content, making them susceptible to microbial action and reduced shelf life [49]. The contents of ash, protein, lipid, carbohydrate, Total Dietary Fiber (TDF), Soluble Dietary Fiber (SDF), Insoluble Dietary Fiber (IDF), total caloric value, ascorbic acid, carotenoids, and phenolic compounds were higher in OP. These compounds act on the fruit peel by delaying the oxidation [50] as well as repelling the presence of herbivores [51] and protecting the fruit from possible mechanical damage [52]. In addition, the higher amount of lipid in OP is due to the presence of D-limonene-producing essential oil vacuoles [53]. 

No difference (*p* > 0.05) was observed between the moisture content in jams. The ash content was higher in OP8 and OP12 jam, while the protein content was higher in OP12 jam in comparison to OP0. The other formulations presented no statistical difference for these two parameters. The addition of OP in the jam reduced carbohydrate content and total energy value, although the difference between the formulations was small. Lipid, TDF, SDF, IDF, ascorbic acid, carotenoids, and phenolic compounds content increased with the addition of OP to the product. Thus, the addition of OP improved the nutritional profile of orange jam. Furthermore, bioactive substances such as ascorbic acid, carotenoids, and phenolic compounds have antioxidant functions, adding potential functional properties to the product. Previous studies have shown that these compounds might reduce the risk of developing some cancers [55,56], diabetes [57], cardiovascular diseases [58], premature aging [59], and inflammation [60]. An increase in the TDF content was also observed in jams OP4 (12.82%), OP8 (33.33%), and OP12 (56.41%), compared to OP0 jam, since OP has a high fiber content (7.86 g 100 g^−1^). 

**Figure 1:**
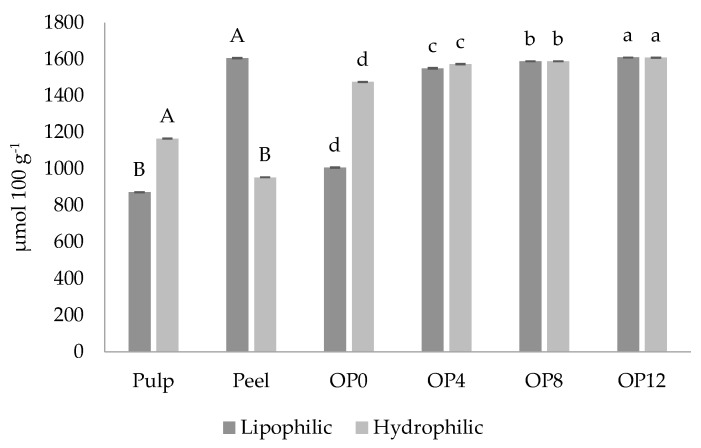
Lipophilic and hydrophilic mean antioxidant capacity of orange pulp and peel, and jam with different orange peel concentrations. Distinct capital letters indicate significant difference with the Student’s *t*-test (*p* < 0.05) for orange pulp and peel; distinct lower case letters indicate significant difference in the Tukey’s test (*p* < 0.05) for jams; the results are reported on a wet basis.

Figure 1 shows the antioxidant capacity of orange pulp and peel, and jam with different OP concentrations. The lipophilic antioxidant capacity of orange peel was higher than that in the pulp (*p* < 0.05), contrary to the evaluation of hydrophilic antioxidant capacity. This effect occurs because the peel has essential oil vacuoles and a greater amount of fat-soluble bioactive compounds, such as carotenoids [37]. Pulp, on the other hand, contains a higher amount of ascorbic acid and water-soluble bioactive compounds as phenols [61,62]. OP addition jams increased lipophilic and hydrophilic antioxidant activities, showing higher activities in OP12. Similar results were observed in fruit peel added jamelao jam (10% to 25%) [63].

## 3. Materials and Methods 

### 3.1. Ethical Issues

The study was conducted in accordance with the Declaration of Helsinki, and the protocol was approved by the Ethics Committee of Midwest State University, protocol number 2.201.325/2017.

### 3.2. Fruit Acquisition

One hundred kg of pear-orange (*Citrus Sinensis*) was used. The fruits were collected in the Center-South region of Paraná State, Brazil, specifically from rural producers of Guarapuava municipality, under the geographical coordinates 25041’12”S latitude, 51038’45”W longitude and altitude of approximately 1100 m. The oranges presented orange peel, smooth surface without imperfections, and were of medium size (169 g). The fruits were sanitized in potable running water, followed by immersion in sodium hypochlorite solution (150 ppm) for 10 min, and washed in potable running water. The peel was extracted manually (1 mm) using a domestic stainless steel grater, yielding 6.5 kg. The peel was still soaked in 26 L of water for 1 h under refrigeration (8 °C) in a plastic container wrapped in polyethylene plastic film. Afterwards, the water was discarded, obtaining a final yield of 14.5 kg of peel. Orange peels were stored under refrigeration (8 °C) until the preparation of the jam.

### 3.3. Jam Formulations

Four orange jam formulations were prepared: OP0 (or standard), OP4, OP8, and OP12 (Orange Peel 0, 4, 8 and 12%, respectively). OP values were defined based on preliminary sensory testing performed with the product (data not shown). The following ingredients were also used: orange pulp (50% in OP0, 46% in OP4, 42% in OP8, 38% in OP12), 49.44% sugar, 0.25% pectin powder (Mago^®^, Cotia, Brazil), and 0.21% citric acid (Synth^®^, Diadema, Brazil). Orange pulp and OP were mixed with sugar at room temperature (22 °C), stirring until homogenization. The mixture was heated in a domestic stove (Atlas^®^, Pato Branco, Brazil) until boiling (103 °C). Pectin was added and cooked for further 2 min. SS content was measured and when they reached the optimal °Brix (63° to 68°), citric acid was added until the pH was adjusted to 3.30 to 3.40. The mixture was cooked for a further 2 min [64]. Hot jams (79–85 °C) were filled in 500 mL glasses. The products were pasteurized at 65 °C for 30 min, cooled to room temperature (22 °C) [65], and stored in a dim environment until further analysis. 

### 3.4. Sensory Evaluation

One hundred and twenty-eight untrained regular jam consumers participated in the survey; they included the students, staff, and teachers of Midwest State University (Guarapuava, Brazil), of both sexes, aged between 18 and 59 years. All subjects gave their informed consent for inclusion in the study. 

The tests were conducted in individual booths with white lighting. Attributes of appearance, aroma, taste, texture and color, and overall acceptance were assessed using a nine-point structured facial hedonic scale ranging from 1 (“very much disliked it”) to 9 (“very much liked it”). Furthermore, a question of purchase intention was applied, structured in a 5-point scale (1–“would not buy it” to 5–“would buy for sure it”) [34]. Consumers got 10 g of each sample in randomized, three-digit coded white disposable plastic cups (50 mL) [66], accompanied by a glass of water for taste cleansing. The formulations were offered in a monadic sequence. The AI was calculated according to the formula: AI (%) = A × 100/B (A = mean grade obtained for the product; B = maximum grade given to the product) [66].

### 3.5. Physicochemical and Nutritional Analysis

Physicochemical determinations were performed in triplicate for orange pulp, OP, and all four jam formulations. All samples were evaluated according to the following parameters and the results were expressed in wet matter: pH measured by a bench pH meter (Tecnopon^®^, MPA-210 model, Piracicaba, Brazil); Aw determined using an Aw analyzer (Novasina^®^, Labswift model, Lachen, Switzerland), operating at 20 °C; SS obtained by direct reading in ABBE bench top refractometer (Bel^®^, RMI/RMT model, Piracicaba, Brazil) [67]. Values were expressed in °Brix; TA using the titration method [67] and results expressed as % citric acid; SS/TA ratio, which was obtained by dividing SS and TA values; RS, NRS and TS evaluated by the Lane-Eynon reductometric method [67]. Results were expressed as g 100 g^−1^; Color, analyzed by the Commission of the International Commission of E’clairage (CIE) L* (lightness), a* (red-green) and b* (yellow-blue), with colorimeter reading (Konica Minolta^®^, Chroma Meter CR 4400 model, Tokyo, Japan) with illuminant D65 and angle 10° [46]. 

The parameters of adhesiveness, viscosity, cohesiveness, gumminess, chewiness, and elasticity were determined by Texture Profile Analysis (TPA). A texturometer (TA.XT Plus^®^, Stable Micro Systems, Godalming, UK), connected to a computer containing Exponet Lite Software (version 4.0.8.0), was used to measure the jam force-time curve using a two-cycle compression. The samples (60 g) were inserted into cylindrical acrylic capsules measuring 5 cm in diameter and 7 cm in height. The samples were compressed by an aluminum cylindrical probe (measuring 3.5 cm in diameter and 3.6 cm in height) at a depth of 5 mm, with a pre-test speed of 1 mm s^−1^, test speed and pos-test speed of 5 mm s^−1^, and 5 g trigger detection force. All the analyzes were performed at room temperature (23 °C). 

For the nutritional composition analysis, the samples were evaluated in triplicate according to the following measurements: moisture content (g 100 g^−1^); ash (g 100 g^−1^); protein (g 100 g^−1^) [67]; Lipid (g 100 g^−1^) [68]; carbohydrate (g 100 g^−1^) by difference method (% Carbohydrate = 100 − (% moisture + % ash + % protein + % lipid + % fiber); total energy value (kcal 100 g^−1^) recommended by Atwater and Woods [69] for lipid (9 kcal g^−1^), protein (4 kcal g^−1^) and carbohydrate–includes dietary fiber (4 kcal g^−1^); total fiber; and insoluble dietary fiber by enzymatic method [67]. The soluble dietary fiber content was calculated by the difference of the total and insoluble dietary fiber results. Ascorbic acid (Vitamin C) was evaluated by the 2.6 dichlorophenolindophenol titration method [67], modified by Benassi and Antunes [70], and the results expressed in mg 100 g^−1^. Phenolic compounds were measured by the Follin-Ciocalteu spectrophotometric method [71]. Readings were performed using a spectrophotometer (Agillent Technologies^®^, Cary 60 UV model, Santa Clara, CA, USA) at 765 nm, and the results were reported as mg of gallic acid equivalent (GAE) 100 g^−1^. Total carotenoids (µg g^−1^) were obtained by spectrophotometric analysis (Agillent Technologies^®^, Cary 60 UV model, Santa Clara, CA, USA) at 450 nm [72]. Antioxidant capacity was evaluated by the ABTS method (2,2-azinobis-[3-ethyl-benzothiazolin-6-sulfonic acid]) in both versions (hydrophilic and lipophilic) [73]. Results were expressed in mmol of Trolox equivalents per gram of sample. The Daily Reference Value (DRV) was calculated for 20 g of jam (1 level tablespoon), based on the recommended daily mean intake values for healthy adults (18 to 59 years) resulting in 2184.9 kcal day^−1^, 202.84 g day^−1^ of carbohydrate, 86.84 g day^−1^ of protein, 15.19 g day^−1^ dietary fiber, and 100.77 mg day^−1^ of ascorbic acid [54].

### 3.6. Statistical Analysis

The results were analyzed using One-Way analysis of variance (ANOVA) test. The means were compared by Tukey’s test and Student’s test at 5% significance level (*p* < 0.05). The Software R was used to perform the statistical calculations.

## 4. Conclusions

An addition of up to 8% of orange peel in orange jam is well accepted by consumers, with sensory acceptance similar to the standard product. Furthermore, OP improved the physicochemical and nutritional profile of the jams by increasing soluble solids, sugars, ash, protein, lipid, dietary fiber, ascorbic acid, carotenoids, phenolic compounds, and antioxidant capacity. However, it reduces carbohydrate and energy content and negatively influences some instrumental color and texture parameters.

The use of food by-products for jam production should be encouraged as it promotes consumers’ access to healthier foods and reduces the negative effects of organic waste disposal on the environment.

## Figures and Tables

**Table 1:** Sensory scores (mean ± standard deviation) of orange jam added with different orange peel contents.

Parameter	OP0	OP4	OP8	OP12
Appearance	7.92 ± 1.05 ^A^	7.85 ± 1.00 ^A^	7.72 ± 1.24 ^A^	7.11 ± 1.33 ^b^
AI (%)	88.00	87.22	85.77	79.00
Aroma	7.61 ± 1.25 ^A^	7.55 ± 1.35 ^A^	7.18 ± 1.11 ^A^	6.63 ± 1.37 ^b^
AI (%)	87.00	85.22	78.88	75.77
Taste	7.71 ± 1.51 ^A^	7.60 ± 1.26 ^A^	7.52 ± 1.27 ^A^	7.07 ± 1.36 ^b^
AI (%)	85.66	84.44	83.55	78.55
Texture	7.75 ± 1.19 ^A^	7.58 ± 1.19 ^A^	7.44 ± 1.24 ^A b^	7.10 ± 1.52 ^b^
AI (%)	86.11	84.22	82.66	78.88
Color	7.82 ± 1.28 ^A^	7.67 ± 1.20 ^A^	7.46 ± 1.24 ^A^	6.67 ± 1.08 ^b^
AI (%)	86.88	85.22	82.88	74.11
Overall Acceptance	7.96 ± 0.96 ^A^	7.89 ± 0.94 ^A^	7.78 ± 0.86 ^A^	7.45 ± 1.12 ^b^
AI (%)	88.44	87.66	86.44	82.77
Purchase Intention	3.80 ± 1.32 ^A^	3.75 ± 1.02 ^A^	3.67 ± 1.13 ^A b^	3.35 ± 0.93 ^b^

Distinct letters on the same line indicate significant difference in the Tukey’s test (*p* < 0.05) for jams; AI: Acceptability Index.

**Table 2:** Physicochemical composition (mean ± standard deviation) of orange pulp and peel, and jam with different orange peel concentrations.

Parameter	Pulp	Peel	OP0	OP4	OP8	OP12
pH	3.97 ± 0.00 ^B^	4.87 ± 0.01 ^A^	3.30 ± 0.03 ^A^	3.31 ± 0.04 ^A^	3.33 ± 0.02 ^A^	3.35 ± 0.05 ^A^
Aw	0.99 ± 0.00 ^A^	0.97 ± 0.00 ^B^	0.75 ± 0.00 ^d^	0.78 ± 0.00 ^c^	0.80 ± 0.00 ^b^	0.82 ± 0.00 ^A^
Soluble Solids (°Brix)	10.13 ± 0.06 ^A^	1.34 ± 0.01 ^B^	63.00 ± 0.00 ^d^	63.50 ± 0.00 ^c^	63.80 ± 0.00 ^b^	64.01 ± 0.00 ^A^
Titratable Acidity (% acid citric)	0.97 ± 0.00 ^A^	0.41 ± 0.00 ^B^	0.57 ± 0.00 ^d^	0.62 ± 0.00 ^c^	0.69 ± 0.00 ^b^	0.76 ± 0.00 ^A^
Soluble Solids/Titratable Acidity Ratio	10.45 ± 0.00 ^A^	3.28 ± 0.01 ^B^	110.53 ± 0.00 ^A^	100.84 ± 0.00 ^b^	91.14 ± 0.00 ^c^	84.85 ± 0.08 ^d^
Reducing Sugars (g 100 g^−1^)	2.60 ± 0.01 ^A^	2.56 ± 0.05 ^A^	16.12 ± 0.02 ^d^	18.61 ± 0.02 ^c^	20.50 ± 0.02 ^b^	23.80 ± 0.00 ^A^
Non-Reducing Sugars (g 100 g^−1^)	9.32 ± 0.06 ^A^	2.78 ± 0.01 ^B^	23.90 ± 0.08 ^c^	24.85 ± 0.02 ^b^	24.91 ± 0.02 ^b^	26.18 ± 0.00 ^A^
Total Sugars (g 100 g^−1^)	11.71 ± 0.08 ^A^	5.60 ± 0.02 ^B^	40.03 ± 0.09 ^d^	43.46 ± 0.00 ^c^	45.41 ± 0.00 ^b^	49.99 ± 0.00 ^A^

Distinct capital letters on the line indicate significant difference in the Student’s *t*-test (*p* < 0.05) for orange pulp and peel; distinct lower case letters on the line indicate significant difference in the Tukey’s test (*p* < 0.05) for jams; the results are reported on a wet basis.

**Table 3:** Color parameters L*, a* and b* (mean ± standard deviation) of orange pulp and peel, and jam with different orange peel concentrations.

Sample	L*	a*	b*
Pulp	41.40 ± 0.73 ^B^	0.34 ± 0.11 ^A^	22.03 ± 1.19 ^B^
Peel	63.50 ± 0.47 ^A^	−1.59 ± 0.23 ^B^	48.49 ± 1.40 ^A^
OP0	57.16 ± 1.75 ^A^	3.96 ± 0.74 ^b^	50.16 ± 1.60 ^A^
OP4	49.77 ± 0.95 ^b^	7.88 ± 0.02 ^A^	43.44 ± 1.63 ^b^
OP8	49.06 ± 1.18 ^b^	6.67 ± 0.13 ^A^	43.73 ± 2.31 ^b^
OP12	42.14 ± 1.98 ^c^	6.68 ± 0.19 ^A^	32.54 ± 2.46 ^c^

Distinct capital letters in the column indicate significant difference in the Student’s *t*-test (*p* < 0.05) for orange pulp and peel; distinct lowercase letters in the column indicate significant difference in the Tukey’s test (*p* < 0.05) for jams; the results are reported on a wet basis.

**Table 4:** Texture parameters (mean ± standard deviation) of jam with different orange peel concentrations.

Parameter	OP0	OP4	OP8	OP12
Adhesiveness	−38.01 ± 1.61 ^A^	−64.68 ± 4.28 ^A^	−90.92 ± 4.77 ^A^	−207.11 ± 43.06 ^b^
Viscosity	0.96 ± 0.03 ^A^	0.97 ± 0.01 ^A^	0.94 ± 0.02 ^A^	0.95 ± 0.02 ^A^
Cohesiveness	0.71 ± 0.03 ^A^	0.76 ± 0.05 ^A^	0.78 ± 0.05 ^A^	0.78 ± 0.09 ^A^
Gumminess	−14.47 ± 2.43 ^d^	102.56 ± 6.99 ^c^	176.86 ± 21.76 ^b^	483.56 ± 14.53 ^A^
Chewiness	−13.92 ± 2.54 ^d^	99.25 ± 7.09 ^c^	167.00 ± 19.40 ^b^	464.21 ± 18.84 ^A^
Elasticity	0.03 ± 0.00 ^b^	0.04 ± 0.01 ^b^	0.08 ± 0.02 ^A^	0.07 ± 0.02 ^A^

Distinct letters on the line indicate significant difference in the Tukey’s test (*p* < 0.05) for jams; the results are reported on a wet basis.

**Table 5:** Nutritional composition (mean ± standard deviation) of orange pulp and peel, and jam with different orange peel concentrations.

Parameter	Pulp	Peel	OP0	DRV (%) *	OP4	DRV (%) *	OP8	DRV (%) *	OP12	DRV (%) *
Moisture (g 100 g^−1^)	89.24 ± 0.02 ^A^	76.25 ± 0.07 ^B^	30.51 ± 0.01 ^A^	ND	30.50 ± 0.02 ^A^	ND	30.52 ± 0.02 ^A^	ND	30.54 ± 0.05 ^A^	ND
Ash (g 100 g^−1^)	0.41 ± 0.01 ^B^	0.89 ± 0.01 ^A^	0.36 ± 0.01 ^B^	ND	0.38 ± 0.01 ^A B^	ND	0.40 ± 0.01 ^A^	ND	0.41 ± 0.01 ^A^	ND
Protein (g 100 g^−1^)	0.83 ± 0.02 ^B^	1.50 ± 0.02 ^A^	1.20 ± 0.01 ^B^	0.24	1.29 ± 0.00 ^A B^	0.25	1.33 ± 0.07 ^A B^	0.26	1.38 ± 0.04 ^A^	0.27
Lipid (g 100 g^−1^)	0.13 ± 0.02 ^B^	1.50 ± 0.01 ^A^	0.05 ± 0.03 ^d^	ND	0.11 ± 0.01 ^c^	ND	0.17 ± 0.05 ^B^	ND	0.22 ± 0.02 ^A^	ND
Carbohydrate (g 100 g^−1^) **	9.47 ± 0.04 ^B^	12.00 ± 0.01 ^A^	66.71 ± 0.25 ^A^	13.34	66.40 ± 0.15 ^A^	13.28	66.02 ± 0.32 ^B^	13.20	65.62 ± 0.19 ^c^	13.12
Energy value (kcal 100 g^−1^)	42.38 ± 0.27 ^B^	67.50 ± 0.04 ^A^	272.09 ± 0.99 ^A^	54.41	271.75 ± 0.87 ^B^	54.35	270.93 ± 0.87 ^c^	54.18	269.98 ± 1.10 ^d^	53.99
Soluble fiber (g 100 g^−1^)	0.34 ± 0.03 ^B^	0.98 ± 0.01 ^A^	0.19 ± 0.04 ^c^	ND	0.20 ± 0.06 ^B c^	ND	0.23 ± 0.07 ^A B^	ND	0.25 ± 0.02 ^A^	ND
Insoluble fiber (g 100 g^−1^)	1.86 ± 0.04 ^B^	6.95 ± 0.01 ^A^	0.98 ± 0.04 ^d^	ND	1.12 ± 0.06 ^c^	ND	1.33 ± 0.02 ^B^	ND	1.59 ± 0.03 ^A^	ND
Total fiber (g 100 g^−1^)	2.35 ± 0.05 ^B^	7.86 ± 0.01 ^A^	1.17 ± 0.06 ^d^	0.23	1.32 ± 0.04 ^c^	0.26	1.56 ± 0.06 ^B^	0.31	1.83 ± 0.05 ^A^	0.36
Ascorbic acid (mg 100 g^−1^)	141.40 ± 0.03 ^B^	190.45 ± 0.00 ^A^	124.83 ± 0.05 ^d^	24.96	130.94 ± 0.08 ^c^	26.18	143.30 ± 0.00 ^B^	28.66	150.12 ± 0.03 ^A^	30.02
Total carotenoid (µg g^−1^)	1.57 ± 0.05 ^B^	17.74 ± 0.13 ^A^	0.87 ± 0.05 ^d^	ND	1.42 ± 0.08 ^c^	ND	2.66 ± 0.02 ^B^	ND	3.39 ± 0.05 ^A^	ND
Phenolic compounds (mg GAE 100 g^−1^)	11.75 ± 0.08 ^B^	31.15 ± 0.06 ^A^	7.08 ± 0.04 ^d^	ND	10.51 ± 0.06 ^c^	ND	11.81 ± 0.06 ^B^	ND	12.73 ± 0.05 ^A^	ND

Distinct capital letters on the same line indicate significant difference in the Student’s *t*-test (*p* < 0.05) for orange pulp and peel; distinct lowercase letters on the line indicate significant difference in the Tukey’s test (*p* < 0.05) for jams; * Daily Reference Value (DRV): nutrients evaluated by the mean DRI [54] are based on a diet of 2.185.8 kcal day^−1^ and a mean portion of 20 g of product; ** Includes dietary fiber; results reported on wet basis; ND: not determined.
